# Sequential low-dose CT thorax scans to determine invasive pulmonary fungal infection incidence after allogeneic hematopoietic cell transplantation

**DOI:** 10.1007/s00277-022-05062-9

**Published:** 2022-12-03

**Authors:** K. Enger, X. Tonnar, E. Kotter, H. Bertz

**Affiliations:** 1 Department of Hematology, Oncology and Stem Cell Transplantation, Faculty of Medicine, Freiburg University Medical Center, Freiburg, Germany; 2 Department of Diagnostic and Interventional Radiology, Freiburg University Medical Center, Freiburg, Germany

**Keywords:** Oncology, Allogeneic hematopoietic stem cell transplantation, Infectious diseases, Fungal infection, Prophylaxis of fungal infection, Incidence of fungal infection, Low-dose CT scans

## Abstract

Invasive fungal disease (IFD) during neutropenia goes along with a high mortality for patients after allogeneic hematopoietic cell transplantation (alloHCT). Low-dose computed tomography (CT) thorax shows good sensitivity for the diagnosis of IFD with low radiation exposure. The aim of our study was to evaluate sequential CT thorax scans at two time points as a new reliable method to detect IFD during neutropenia after alloHCT. We performed a retrospective single-center observational study in 265/354 screened patients admitted for alloHCT from June 2015 to August 2019. All were examined by a low-dose CT thorax scan at admission (CT t_0_) and after stable neutrophil recovery (CT t_1_) to determine the incidences of IFD. Furthermore, antifungal prophylaxis medications were recorded and cohorts were analyzed for statistical differences in IFD incidence using the sequential CT scans. In addition, IFD cases were classified according to EORTC 2008. At CT t_0_ in 9.6% of the patients, an IFD was detected and antifungal therapy initiated. The cumulative incidence of IFD in CT t_1_ in our department was 14%. The use of Aspergillus-effective prophylaxis through voriconazole or posaconazole decreased CT thorax t_1_ suggesting IFD is statistically significant compared to prophylaxis with fluconazole (5.6% asp-azol group vs 16.3% fluconazole group, *p* = 0.048). In 86%, CT t_1_ was negative for IFD. Low-dose sequential CT thorax scans are a valuable tool to detect pulmonary IFDs and guide antifungal prophylaxis and therapies. Furthermore, a negative CT t_1_ scan shows a benefit by allowing discontinuation of antifungal medication sparing patients from drug interactions and side effects.

## Background

Patients undergoing allogeneic hematopoietic cell transplantation (alloHCT) are at risk for invasive fungal disease (IFD), especially during neutropenia [1, 2]. Due to the high mortality [1, 3] prophylaxis, early diagnosis and therapy are of highest importance [4, 5]. The main pathogens are non-albicans Candida and Aspergillus [2, 6, 7]. In 90% of cases, IFD presents with an affection of the lungs [8]. Early diagnosis is complicated by late, nonspecific symptoms [9]. Computed tomography (CT) of the thorax is established for early diagnosis of pulmonary IFD. Studies have shown that screening by CT thorax reduces the time to diagnosis and thus leads to an improvement in outcome [10–14]. A combination with serum markers (galactomannan, ß-D-glucan), bronchoscopy with bronchoalveolar lavage (BAL), biopsies with culture and histology increases the sensitivity and specificity of the diagnosis and is currently the gold standard [15, 16]. These diagnostic criteria are also reflected in the EORTC/MSG classifications, which were developed for the comparability of studies. It divides the IFD into possible, probable, and proven cases [17].

Antifungal prophylaxis is recommended in patients during neutropenia (pre-engraftment period) after alloHCT. With a low incidence of Aspergillus, Aspergillus-targeted diagnostics (CT thorax, serum markers), and Aspergillus-targeted therapy (empiric/preemptive), fluconazole is currently the prophylactic drug of choice [18]. Important for the decision for a prophylactic drug is the patient’s risk profile [19], potential toxicity and interactions with other drugs [4], cost [20], and spectrum of activity and efficacy [21]. Because the predominant pathogens change over time and may vary locally [8], knowledge of the local incidence of a center is recommended [18].

Routine screening for IFD by CT thorax on admission (CT t_0_) and after neutrophil regeneration after alloHCT (CT t_1_) is not yet established in current guidelines. Baseline CT (CT t_0_) offers the advantage of identifying patients who require treatment and not prophylaxis [22]. After the stable neutrophil recovery signs of IFD such as cavity formation as well as the air-crescent sign become visible [23–27], this justifies the performance of a CT t_1_. In the case of a negative CT t_1_, discontinuation of the medication can be considered [28]. The most frequent point of criticism of this procedure is the radiation exposure with its possible consequential long-term damage [29]. By using low-dose CT instead of standard CT, reduction of radiation exposure (approx. 1–4 mSv instead of approx. 7 mSv) can be achieved with good diagnostic quality [29–31]. It is superior to chest X-ray (radiation exposure 0.06–0.25 mSV [32]) for the diagnosis of IFD [31, 33].

We examined IFD incidence and different antifungal strategies for the first time using sequential low-dose CT thorax at admission (CT t_0_) and CT thorax after neutrophilic regeneration (CT t_1_).

## Methods

### Patients

During the study period from June 2015 to August 2019, 354 consecutive patients undergoing alloHCT at Freiburg University Hospital were included in the study. 98 patients had to be excluded. Reasons for exclusion were age below 18 years, IFD-positive CT t_0_, the absence of a CT t_1_, and the lack of Helsinki informed consent. Overall, 265 patients could be evaluated for this retrospective study.

### Data collection

Data collection was obtained from registry data and electronic medical records. Low-dose CT thorax was evaluated in all patients at admission for alloHCT (CT t_0_) and after stable regeneration of neutrophil granulocytes (CT t_1_). The latter was defined by a neutrophil granulocyte count of > 500/μl on 3 consecutive days. For this purpose, the text from the radiologists were evaluated and the CT scans were classified as IFD positive or negative. For classification into “positive” or “negative,” IFD-suggestive signs like the Halo sign, reversed Halo sign, air-crescent sign, and tree-in-bud sign were used. Patient characteristics (Table 1) were evaluated for all patients. Antifungal prophylaxis was recorded.

**Table 1:** Patients characteristics

		IFD in CT t_1_	%	No IFD	%	Total	%
		37		228		265	
Sex	f	13	35.1	88	38.6	101	38.1
	m	24	64.9	140	61.4	164	61.9
Age (years)	Range	35.2–73.3		18.7–79.1		18.7–79.1	
	Median	56.6		62.2		60.6	
Underlying disease	Myeloid	29	78.4	167	73.3	196	74.0
	Lymphatic	6	16.2	53	23.3	59	22.3
	Other	2	5.4	8	3.5	10	3.7
Donor characteristics	MRD	7	18.9	45	19.7	52	19.6
	MUD	19	51.4	121	53.1	140	52.9
	mMRD	3	8.1	18	7.9	21	7.9
	mMUD	8	21.6	44	19.3	52	19.6
GvHD prophylaxis	Cyclosporine + MMF + Cyclophosphamide	3	8.1	26	11.4	29	10.9
	Cyclosporine + MMF	5	13.5	41	18.0	46	17.4
	Cyclosporine + MMF + ATG	28	75.7	156	68.4	184	69.4
	other	1	2.7	5	2.2	6	2.3
Transplantation number	1	36	97.3	208	91.2	244	92.1
	2 or more	1	2.7	20	8.8	21	7.9
Conditioning regime	NMA/RIC	35	94.6	199	87.3	234	88.3
	MA	2	5.4	29	12.7	31	11.7
Remission status	CR/CP	5	13.5	55	24.1	60	22.6
	Not CR/CP	32	86.5	173	75.9	205	77.4
Pulm. IFD in patient history	Yes	3	8.1	11	4.8	14	5.3
	No	34	91.9	217	95.2	251	94.7
Other IFD in patient history	Yes	0	0.0	1	0.4	1	0.4
	No	37	100.0	227	99.6	264	99.6
Classification of IFD according to EORTC criteria	None	0	0.0	213	93.4	213	80.4
	Possible	25	67.6	7	3.1	32	12.0
	Probable	10	27.0	4	1.8	14	5.3
	Proven	2	5.4	4	1.8	6	2.3

*Abbreviations: ATG*, Anti-thymocyte globulin; *CP*, Chronic phase; *CR*, complete remission; *CT*, Computed tomography; *EORTC*, European Organization for Research and Treatment of Cancer; *f*, Female; *GvHD*, Graft versus Host Disease; *RIC*, Reduced intensity conditioning; *m*, Male; *MA*, Myeloablative; *MMF*, Mycophenolate mofetil; *mMRD*, Mismatched related donor; *MRD*, Matched related donor; *mMUD*, Mismatched unrelated donor *MUD*, matched unrelated donor; *NMA*, Non-myeloablative.

Additionally, all patients were classified into possible, probable, and proven IFD according to the EORTC criteria 2008 [17] as it is the most common classification for IFD, especially for comparability of studies. EORTC 2008 divides IFDs into three categories with increasing probability of having an IFD. A possible IFD combines a positive clinical criterion (IFD suspicious CT scan, tracheobronchitis suspicious bronchoscopy, sinonasal infection suspicious CT scan, CNS infection suspicious brain imaging, or signs of a disseminated candidiasis) with a positive host factor. A probable IFD is defined by the combination of a positive clinical criterion, a positive host factor, and a positive mycological criterion. An IFD is classified as proven if a fungus is detected in a sterile probe [17].

Furthermore, changes in therapy were recorded. Changes in therapy were categorized into empirical, preemptive, and targeted therapy [34]. Empirical therapy was defined as fever persistence longer than 3 days despite broad-spectrum antibiotics. Preemptive therapy was defined as positive diagnostic measures, e.g., positive serological testing, and targeted treatment was used after confirmed fungal infection [34].

### Statistical analysis

The primary endpoint, incidence of IFD in sequential low-dose CT thorax scans CT t_0_ and CT t_1_ was descriptively presented and expressed in percent. The secondary endpoint was the IFD incidence among the different prophylactic drugs. The incidences of each drug were reported descriptively and the most common drugs were compared. Fisher exact test was used to evaluate statistical differences. Chi-square test was used in cases of more than two manifestations of the variables. The statistical significance level was set at a *p*-value < 0.05. The odds ratio and 95% confidence interval were reported. Logistic regression was performed to adjust the result for possible confounders. Possible confounders included type of conditioning, type of graft-versus-host disease (GvHD) prophylaxis, and remission status pre-transplant.

## Results

### Patient characteristics

The basic characteristics of the patients are listed in Table 1.

### Primary endpoint

At CT t_1_ 37/265 patients were diagnosed with IFD, despite all 265 patients receiving antifungal prophylaxis from the beginning of the conditioning therapy. Thus, the cumulative incidence of IFD via sequential CT thorax scans was 14%.

According to the EORTC 2008 criteria, 32 patients (12.1%) were classified as possible, 14 patients (5.3%) as probable, and 6 patients (2.3%) as proven.

Of 228 patients with negative CT t_1_ scan, 7 patients (3.1%) were classified as possible, 4 patients (1.7%) as probable, and 4 patients (1.7%) as proven according to EORTC criteria.

We detected 33/354 (9,3%) patients with IFD-positive CT t_0_. Among these 33 patients, 22 patients (67%) had a pre-existing IFD. In the other 11 patients (33%), the initial diagnosis of IFD was made on basis of CT t_0_. On closer analysis of these patients, the risk factor not CR/CP is particularly striking: 25 of 33 patients (76%) were not in complete remission or not in a chronic phase at the time of CT t_0_.

### Secondary endpoint

#### Prophylaxis

Fluconazole prophylaxis was administrated to 171 patients (64.5%), their IFD incidence was 16.4% in CT t_1_. 54 patients (20.4%) received Aspergillus-effective prophylaxis with posaconazole or voriconazole (asp-azol group). Both azoles were grouped together because of a similar pathogen spectrum [35]. IFD incidence in the CT thorax was considerably lower in this cohort (5.6%). Fewer patients received an IFD prophylaxis with liposomal amphotericin B (8.8% in the cohort; 13% IFD incidence) or caspofungin (1.5% in the cohort; 16.7% IFD incidence). In 21 cases, multiple prophylactic drugs were used sequentially. These combinations did not occur for infectious reasons but were mostly due to drug interactions. The most common prophylaxis groups were compared.

Comparing fluconazole prophylaxis (16.4% IFD incidence) with all other antifungal prophylaxis (9.6% IFD incidence), there was no statistically significant difference in IFD incidences in CT (16.4% vs. 9.6%, *p* = 0.1265).

Prophylaxis in the asp-azol group (5.6% IFD incidence) was compared with prophylaxis with fluconazole, caspofungin, or liposomal amphotericin B (16.1% IFD incidence). With a *p*-value by Fisher’s exact test of 0.048, this difference was statistically significant (CI (RR): [0.110; 1.08]). With a number needed to treat of 9.54, this result showed a clinical significance.

As fluconazole prophylaxis dominated the comparison group, this group was compared individually with the asp-azol group.

Prophylaxis with fluconazole (IFD incidence 16.4%) was compared to the asp-azol cohort (IFD incidence 5.6%) regarding statistical differences of IFD incidence. With a *p*-value by Fisher’s exact test of 0.044, this difference in incidence was statistically significant (CI (RR) [0.933;9.315]). The number needed to harm was 9.26.

Logistic regression showed no significant influence of confounders on the probability of occurrence of IFD (Table 2). With a *p*-value of 0.037 fluconazole prophylaxis is a significant independent risk factor for the development of IFD in CT thorax after adjusting for confounders. Fluconazole demonstrated significant inferiority in the prophylaxis of IFD compared to posaconazole/voriconazole prophylaxis. Logistic regression showed no significant differences in all our results, summarized in Table 3.

**Table 2:** Summary of logistic regression with confounders

		*p*-value	OR	95% CI (OR)	SE
Flu//other prophylaxis	Adjusted value	0.088	2.02	[0.900;4.53]	0.41
Posa/Vori (asp-azolgroup)//other prophylaxis	Adjusted value	0.048	0.288	[0.083;0.990]	0.63
Flu//Posa/Vori (asp-azolgroup)	Adjusted value	0.037	3.784	[1.086;13.173]	0.64

*Abbreviations: asp-azol*, Aspergillus effective azole; *Flu*, fluconazole; *Posa*, posaconazole; *Vori*, voriconazole.

**Table 3:** Logistic regression results, fluconazole prophylaxis compared to posaconazole/voriconazole prophylaxis and confounders

Coefficients	Log OR	SE	*p*-value
Fluconazole prophylaxis	1.331	0.646	0.037
NMA	0.867	0.777	0.265
Cyclosporine + MMF	− 0.485	0.586	0.408
Cyclosporine + MMF + Cyclophosphamide post- transplant	− 0.332	0.666	0.618
Other	− 15.063	1061.776	0.989
No CR/CP	0.764	0.571	0.181

*Abbreviations: CP*, chronic phase; *CR*: complete remission; *MMF*, Mycophenolate mofetil; *NMA*, nonmyeloablative; *OR*, odds ratio; *SE*, standard error.

#### Changes of therapy

Prophylaxis only was received by 142 patients (53.6%). Empirical therapy was used in 108 patients (40.8%). Preemptive therapy was used in 19 patients (7.2%) and targeted treatment was used in 5 patients (1.9%). Because of 9 cases of multiple classifications, the addition of these values exceeds 100%.

With 102 patients (59.6%), more than half of the patients receiving fluconazole prophylaxis needed a change of therapy. In comparison, only 21 patients (22.3%) receiving an Aspergillus-effective prophylaxis needed a change of therapy.

Focusing on the most common change of therapy, the empirical therapy, we saw that of the 108 patients (40.8%) that underwent empirical escalation, only 18 patients (16.7%) had an IFD detected in CT t_1_. In the other 90 patients (83.3%), no IFD signs were detected in CT t_1_.

## Discussion

In this study, 265 patients were evaluated for the incidence of IFD after alloHCT at Freiburg University Hospital between June 2015 and August 2019. Using sequential low-dose CT scans on admission and after neutrophil regeneration following alloHCT for the first time, this retrospective study showed a cumulative incidence of IFD of 14% (37/265 patients).

According to the EORTC criteria (version 2008), 12.1% were classified as possible, 5.3% as probable, and 2.3% as proven, resulting in a cumulative incidence of 19.7%.

The difference between these results is due to the diagnostic breadth that classifies IFDs into possible (host factor plus clinical criterion), probable (host factor plus clinical criterion plus a mycological criterion), or proven (positive culture from sterile material) IFDs. By the nature of the patient collective of this study, all had a positive host factor. Besides the CT thorax signs mentioned previously, positive clinical criteria could be established with a tracheobronchitis suspicious bronchoscopy, sinonasal infection suspicious CT scan, CNS infection suspicious brain imaging, or signs of a disseminated candidiasis [17].

Comparing our findings with previous results, our study population, with an average age of 60.6 years, was older than the cohorts of the comparative studies [2, 36, 37]. In addition, only 22.6% of patients were in CP1/CR1 at the time of admission for alloHCT. 80.5% of patients received stem cells from an unrelated or non-HLA-identical donor. These three characteristics, which are associated with an increased risk of IFD, were overrepresented in the study population [2, 19, 36]. Furthermore, three risk factors were underrepresented in the study population (myeloablative conditioning, intermediate risk in the intensity of GVHD prophylaxis, IFD in patient history) [4, 38].

Comparing the cases of proven and probable IFD, Martino et al. found an annual incidence of 14% compared with 19.6% in our work [2]. However, the cohort of the study by Martino et al. consisted of significantly younger patients, and only MRD (matched related donor) transplants were included. Due to these differences, there is a lower IFD risk in the cohort of Martino et al. [19, 38].

Marr et al. showed an annual incidence for proven/probable Aspergillosis after MRD transplantation of 7.3% and after mMUD/mMRD and MUD of 10.5% [36]. However, the cohort in this study and the cohort in our work differ significantly. In the cohort of Marr et al., there were patients with higher IFD risk (patients with myeloablative conditioning) as well as patients with lower IFD risk (younger patients, patients in CP/CR) compared to our study [35]. Previous studies demonstrated that these features, in addition to patient age, remission status CP/CR, and myeloablative conditioning, significantly influence the risk of IFD [19, 38].

In contrast, the study by Post et al. showed an incidence of 23% for all possible, probable & proven invasive Aspergillosis [37]. The patient population was older and had a less favorable donor profile, going along with an increased IFD risk [38].

Hence, both the cumulative incidence of IFD detected by CT thorax (14%) and the cumulative incidence according to EORTC criteria (19.6%) are comparable to previous studies and should be interpreted in the context of a heterogeneous patient group.

The EORTC criteria are better suited to compare studies and thus different prophylactic strategies. It should be mentioned that a total of 6.6% of patients with possible, probable, or proven IFD were not detected by sequential CT examinations. Of these 15 patients, 4 patients (1.7%) had proven IFD. At this point, we want to clarify that the aim of this work concerns the detection of invasive pulmonary fungal infections by sequential CT scans. Non-pulmonary invasive fungal infections cannot, of course, be diagnosed by sequential CT thorax scans, but stood out in the evaluation because they were diagnosed according to EORTC criteria.

As good as the EORTC criteria are for comparing studies, the clinical utility is questionable. In particular, in the phase between CT t_0_ and CT t_1_ during alloHCT, it is not feasible to perform the necessary diagnostics to diagnose a proven IFD: due to thrombocytopenia, there is a high risk of bleeding during necessary bronchoscopies and possible punctures for biopsy(15, 39). In addition, there is a high risk for iatrogenic infections because the regeneration of white blood cells has not yet occurred. Thus, diagnosis of possible and probable IFDs is more likely to occur. Due to these circumstances, overdiagnosis of actual IFDs is conceivable, which may result in unnecessarily long antifungal prophylaxis and therapy. For this reason, the focus of this work was to determine the incidence of IFD using sequential low-dose CT thorax scans only; to our knowledge, such an approach has not been followed yet.

Even though it is not currently part of the guideline, the results of this study encourage the implementation of a CT thorax scan on admission to alloHCT (CT t_0_): 9.6% of 354 patients had to be excluded from our study because of IFD-specific CT thorax morphology in CT t_0_ as incidental finding. If CT t0 is not performed, these patients won’t be detected and would receive prophylactic therapy instead of therapeutic antifungal medication before undergoing conditioning and subsequently alloHCT. Stemler et al. demonstrated that CT t_0_ is already becoming standard-of-care in 43 different countries in high-risk hematology patients, including patients admitted for alloHCT [22]. Due to the high risk of IFD in this patient collective [1, 2] and the associated increased mortality [1, 3], we recommend that CT t_0_ should be included in the current guidelines.

In addition, our study analyzed the importance of routine CT t_1_ to detect new IFD. By doing so, the cumulative incidence of IFD based on CT morphologic changes for the period from conditioning to regeneration of neutrophil granulocytes after alloHCT could be calculated for the first time yet: the cumulative IFD incidence was 14%. Numerous preliminary studies have already demonstrated that early detection of IFD improves the outcome [11, 13, 28, 40]. By routinely performing CT t_1_, IFDs can be diagnosed and treated more rapidly, allowing potentially severe and fatal courses to be treated early. Moreover, CT t_1_ allows evaluating the prophylaxis or therapy (empiric, preemptive, or targeted) carried out so far: new or progressive IFD-specific CT thorax morphologies lead to an early change of the antifungal therapy.

If we focus on the 86% of patients with negative CT t_1_, we identify an advantage for this group as well: regardless of whether the patients received prophylaxis, preemptive, empirical or targeted antifungal medication, on the basis of negative CT t_1_ discontinuation should be considered. This reduces costs, side effects, and potential interactions [28]. Especially in a strategy of early empirical escalation, it is important to identify a strategy to end the therapy. In our cohort, 108 patients (40.8%) underwent early empirical escalation. Of these, only 18 patients (16.7%) had IFD detected in CT t_1_. Thus, in the other 90 patients (83.3%), antifungal therapy could be stopped after CT t_1_.

Considering these results, we would like to encourage the discussion of implementing CT t_1_ following alloHCT in the current guidelines.

We would particularly like to emphasize the benefits of low-dose CT for this purpose. Radiation exposure can be kept low at 1–4 mSv while maintaining a good diagnostic quality [30, 31, 33, 41]. With ultra-low dose CT (0.25–0.6 mSV), radiation exposure could even be reduced to the level of a chest X-ray examination [30, 41].

Further, we evaluated the former prophylaxis strategy using fluconazole. Due to the very similar pathogen spectrum, the Aspergillus-active azoles voriconazole and posaconazole were evaluated in one group (asp-azol group). With this study, we have confirmed that IFD incidence was statistically significantly lower in the asp-azol group than in the rest of the cohort (fluconazole, L-AmB, and caspofungin). Even after adjusting for confounders by logistic regression, this significant difference is evident. We calculated a number needed to treat of almost 10.

It is well demonstrated that voriconazole/posaconazole leads to an inhibition of CYP 450 isoenzyme 14α-demethylase with consequently increased levels of immunosuppressant drugs with potentially serious side effects [42, 43]. This interaction is much more pronounced with posaconazole/voriconazole than with fluconazole [44]. In addition, there are more toxicities under the asp-azol therapy [45, 46]. Discontinuation of posaconazole or voriconazole is accompanied by lowered immunosuppressant blood levels and therefore tapering is recommended [42].

Comparing posaconazole with fluconazole/itraconazole in neutropenic patients, O'Sullivan et al. showed a significant reduction in proven and probable IFD incidence of 6%, which was associated with both an increase in overall survival and remarkably a reduction in total cost ($3900 vs $4500). Thus, despite a higher drug price, prophylaxis with posaconazole is economically superior to prophylaxis with fluconazole/itraconazole [47].

Considering the statistically significant better efficacy of voriconazole/posaconazole prophylaxis regarding IFD occurrence in CT thorax, the findings in previous studies (45–47), and being aware of the possible side effects of the asp-azol drugs, we would encourage each transplant center to reevaluate their anti-fungal prophylaxis strategy.

In conclusion, sequential low-dose CT thorax scans should be routinely performed upon admission and after neutrophil recovery to optimize alloHCT patients’ IFD care. While the CT t_0_ helps to start a necessary therapy that otherwise would not have been started or to adjust an existing therapy, the CT t_1_ helps to stop unnecessary therapies or prophylaxes.
